# The Role of Programmed Cell Death 1/Programmed Death Ligand 1 (PD-1/PD-L1) Axis in Sepsis-Induced Apoptosis

**DOI:** 10.3390/medicina60071174

**Published:** 2024-07-19

**Authors:** Oana Coman, Bianca-Liana Grigorescu, Adina Huțanu, Anca Bacârea, Anca Meda Văsieșiu, Raluca Ștefania Fodor, Florin Stoica, Leonard Azamfirei

**Affiliations:** 1 Department of Simulation Applied in Medicine, University of Medicine, Pharmacy, Science and Technology “George Emil Palade”, 540142 Targu Mures, Romania; oana.coman@umfst.ro; 2 Department of Anaesthesiology and Intensive Therapy, University of Medicine, Pharmacy, Science and Technology “George Emil Palade”, 540142 Targu Mures, Romania; raluca.fodor@umfst.ro (R.Ș.F.); leonard.azamfirei@umfst.ro (L.A.); 3 Department of Laboratory Medicine, University of Medicine, Pharmacy, Science and Technology “George Emil Palade”, 540142 Targu Mures, Romania; adina.hutanu@umfst.ro; 4 Center for Advanced Medical and Pharmaceutical Research, Immunology, University of Medicine, Pharmacy, Science and Technology “George Emil Palade”, 540142 Targu Mures, Romania; 5 Department of Pathophysiology, University of Medicine, Pharmacy, Science and Technology “George Emil Palade”, 540142 Targu Mures, Romania; anca.bacarea@umfst.ro; 6 Department of Infectious Disease, University of Medicine, Pharmacy, Science and Technology “George Emil Palade”, 540142 Targu Mures, Romania; anca-meda.georgescu@umfst.ro; 7 Clinic of Internal Medicine II, Emergency County Hospital, 540136 Targu Mures, Romania; stoicaflorin953@gmail.com

**Keywords:** sepsis, septic shock, programmed cell death, apoptosis, lymphocytes, CD4+ lymphocytes, CD8+ lymphocytes, PD-1/PD-L1 axis, SOFA, APACHE II

## Abstract

*Background and Objectives*: Sepsis involves a dysregulated host response, characterized by simultaneous immunosuppression and hyperinflammation. Initially, there is the release of pro-inflammatory factors and immune system dysfunction, followed by persistent immune paralysis leading to apoptosis. This study investigates sepsis-induced apoptosis and its pathways, by assessing changes in PD-1 and PD-L1 serum levels, CD4+ and CD8+ T cells, and Sequential Organ Failure Assessment (SOFA) and Acute Physiology and Chronic Health Evaluation (APACHE II) severity scores. *Materials and Methods*: This prospective, observational, single-centre study enrolled 87 sepsis patients admitted to the intensive care unit at the County Emergency Clinical Hospital in Târgu Mureș, Romania. We monitored the parameters on day 1 (the day sepsis or septic shock was diagnosed as per the Sepsis-3 Consensus) and day 5. *Results*: Our study found a statistically significant variation in the SOFA score for the entirety of the patients between the studied days (*p* = 0.001), as well as for the studied patient groups: sepsis, septic shock, survivors, and non-survivors (*p* = 0.001, *p* = 0.003, *p* = 0.01, *p* = 0.03). On day 1, we found statistically significant correlations between CD8+ cells and PD-1 (*p* = 0.02) and PD-L1 (*p* = 0.04), CD4+ and CD8+ cells (*p* < 0.0001), SOFA and APACHE II scores (*p* < 0.0001), and SOFA and APACHE II scores and PD-L1 (*p* = 0.001 and *p* = 0.01). On day 5, we found statistically significant correlations between CD4+ and CD8+ cells and PD-L1 (*p* = 0.03 and *p* = 0.0099), CD4+ and CD8+ cells (*p* < 0.0001), and SOFA and APACHE II scores (*p* < 0.0001). *Conclusions*: The reduction in Th CD4+ and Tc CD8+ lymphocyte subpopulations were evident from day 1, indicating that apoptosis is a crucial factor in the progression of sepsis and septic shock. The increased expression of the PD-1/PD-L1 axis impairs costimulatory signalling, leading to diminished T cell responses and lymphopenia, thereby increasing the susceptibility to nosocomial infections.

## 1. Introduction

Medical records dating back to 1000 BC note instances of infectious diseases in humans. Despite advances in medicine, sepsis ranks as the foremost cause of mortality among critically ill patients, with a mortality rate reaching as high as 46.4%, especially in aging patients with multiple comorbidities [1,2,3]. 

The dysregulated host response in sepsis is characterized by intertwined states of immunosuppression and hyperinflammation. According to the Third International Consensus, the updated definition of sepsis and septic shock encompasses these phenomena more comprehensively than previously acknowledged. The up-to-date definition of septic shock describes profound circulatory, cellular, and metabolic abnormalities associated with a greater risk of mortality than sepsis [4].

A novel term associated with sepsis is immunosenescence, affecting both innate and acquired immunity and presenting characteristics like sepsis, such as lymphocytopenia and immunosuppressive cell proliferation [5,6,7].

Initially, sepsis manifests with the release of pro-inflammatory factors, such as TNF-a, IL-1b, IL-6, IL-12, and IL-18, the continuous activation and dysfunction of the immune system, followed by persistent immune paralysis [8]. The pathophysiology of sepsis implies the presence of a “cytokine storm” acting as a catalyst for immunosenescence [9,10]. The immunosuppression acquired in septic patients encompasses a variety of cell types and features. It is associated with heightened apoptosis of immune cells, the exhaustion of T cells, cellular reprogramming through epigenetic modifications, and the diminished expression of activating cell-surface molecules. Alterations leading to immune suppression have been linked to the heightened vulnerability of sepsis patients to secondary infections, frequently instigated by opportunistic pathogens and viral reactivation. Apoptosis predominantly affects CD4+ T cells, CD8+ T cells, B cells, natural killer (NK) cells, and follicular dendritic cells. Both death receptor- and mitochondrial-mediated pathways play a role in this process [9,11,12,13].

In preclinical sepsis models, inhibiting or preventing apoptosis shows protective effects, emphasizing its functional significance. Compromised autophagy may also contribute to immunosuppression, by failing to remove redundant or dysfunctional cellular components [9,14].

Among patients with sepsis, T cell immune dysfunction stands out as a crucial factor contributing to significant impairment of overall immune function. Timely identification of the T cell immune status in sepsis patients, coupled with enhanced supportive therapy, has the potential to mitigate mortality rates [15].

Immune checkpoint pathways are intrinsic components of the immune system that regulate immune responses in normal physiological conditions. The immune checkpoint involving programmed cell death protein 1 (PD-1) and programmed death ligand 1 (PD-L1) plays an essential role in inhibiting activation signals induced by the T cell receptor. Moreover, the interaction between PD-L1 and PD-1 systematically suppresses the immune system across various cell types [16,17].

Survivors of initial sepsis episodes frequently experience nosocomial infections caused by organisms that are typically non-pathogenic in individuals with a healthy immune system. Additionally, the reactivation of latent viruses is common. The PD-1 and interleukin 7 (IL-7) pathway has emerged as a significant mechanism inhibiting T cell function, thereby being linked to late sepsis and subsequent immunosuppression in individuals who survive the initial septic episode [4]. The aftermath of sepsis survival is associated with enduring, chronic consequences on host protective immunity [18].

Considering the research spotlight on finding early, valuable, and cost-effective predictors for sepsis outcomes, this study aimed to investigate sepsis-induced apoptosis and its pathways, assessing the changes in PD-1 and PD-L1 serum values, CD4+ T cells, CD8+ T cells, and Sequential Organ Failure Assessment (SOFA) and Acute Physiology and Chronic Health Evaluation (APACHE II) severity scores, on day 1 and day 5, in patients presenting with either sepsis or septic shock.

## 2. Materials and Methods

### 2.1. Study Design and Patient Characteristics

This prospective, observational, single-centre study enrolled 87 sepsis patients admitted to the intensive care unit (ICU) at the County Emergency Clinical Hospital in Târgu Mureș, Romania, between July 2021 and March 2023.

Ethical approval for this study was obtained from the Ethics Committee at the University of Medicine, Pharmacy, Science, and Technology ‘G.E. Palade’ in Târgu Mureș (Mureș, Romania; approval no. 1425/01.07.2021), and the research adhered to the principles of the Helsinki Declaration.

The participants included individuals aged 18 and above, diagnosed with sepsis, according to the Sepsis-3 Consensus criteria [4]. The exclusion criteria encompassed current cancer with chemotherapy or radiation therapy, ongoing treatment with corticosteroids or immunosuppressive medication, and evidence of autoimmune disorders.

Informed consent for study participation and data publication was obtained from each patient or their legal guardian.

### 2.2. Evaluated Parameters

The parameters under examination included age, gender, complete blood count (CBC), Th cells (CD4+), Tc cells (CD8+), programmed cell death 1 (PD-1), and programmed death ligand 1 (PD-L1), as well as the SOFA score and APACHE II score. All these factors were assessed on both the first and fifth day after confirmation of either sepsis or septic shock in the intensive care unit (ICU), according to the Sepsis-3 Consensus criteria [4].

Venous blood samples were collected from each study participant, via peripheral puncture using 10 mL syringes. For leukocyte subset identification, blood was collected in K2 EDTA tubes and sent for processing. For PD-1/PD-L1 expression quantification, blood was collected in clot activator tubes, centrifuged for 15 min at 2588 revolutions per minute, aliquoted, and stored at −80 °C in 1.5 mL Eppendorf tubes, until all the patients were recruited.

For CBC and immunophenotyping, venous blood samples were collected in K2 EDTA tubes. The identification of leukocyte subsets was performed using a BD FACSCalibur™ flow cytometer analyzer (Becton, Dickinson and Company, Franklin Lakes, NJ, USA) and the interpretation of the data was performed with a BD CellQuest™ Pro (Becton, Dickinson and Company, Franklin Lakes, NJ, USA). The subsets of leukocytes were expressed as % of the total peripheral blood mononuclear cells (PBMCs). We gated the lymphocytes using a side scatter/CD45 density plot.

The following combination of monoclonal antibodies conjugated to specific fluorochromes, using BD^®^ Biosciences reagents, was used:

- CD4/CD8/CD3 (BDTritest, Cat. No. 342414);
- CD3/CD16 + CD56/CD45/CD19 (BD Multitest, Cat. No. 342416).

The levels of cytokine/target proteins in terms of PD-1 and PD-L1 were assessed using an ELISA protocol. Commercial kits designed for detecting recombinant and natural human programmed cell death 1 and programmed death ligand 1 were employed, following the manufacturer’s instructions (EIAab Science Inc, Wuhan, China), and were processed using automated ELISA equipment (Dynex DSX, Dynex Technology USA, Chantilly, Virginia, VA, USA). Blood samples were collected via venous puncture using clot activator tubes, then centrifuged for 15 min at 2588 revolutions per minute. The serum was subsequently aliquoted and stored at −80 °C in 1.5 mL Eppendorf tubes, until all the patients were recruited. The detection range for PD-1 was 0.156–10 ng/mL, the sensitivity was <0.067 ng/mL, and the coefficient of variation was ≤4.2% for the intra-assay and ≤8.7% for the inter-assay precision. For PD-L1, the detection range was 0.312–20 ng/mL, the sensitivity was <0.098 ng/mL, and coefficient of variation was ≤6% for the intra-assay and ≤7.9% for the inter-assay precision.

### 2.3. Statistical Analysis

The information was inputted into MS Excel (Microsoft® Excel® for Microsoft 365 MSO (Version 2406 Build 16.0.17726.20078, Microsoft Corporation, Redmond, WA, USA). Subsequent statistical analyses, encompassing descriptive and inferential processing, were conducted using GraphPad Prism version 8.4.3 (686), released on 10 June 2020 (GraphPad Software, San Diego, CA, USA). Descriptive statistics included the computation of the means or medians, with the corresponding confidence intervals. For data exhibiting normal distribution, the mean was determined, while for non-Gaussian distributions, the median and the interquartile range were calculated. For paired data, we used either Student’s t-test or the Wilcoxon test, depending on whether the distribution was Gaussian or non-Gaussian. For receiver operating characteristic (ROC) analysis, RStudio version 2023.12.1+402, released on 29 January 2024 (Posit, PBC, Boston, MA, USA), was used. Moreover, *p* ≤ 0.05 was considered to indicate a statistically significant difference.

## 3. Results

### 3.1. Population Analysis

**Figure 1:**
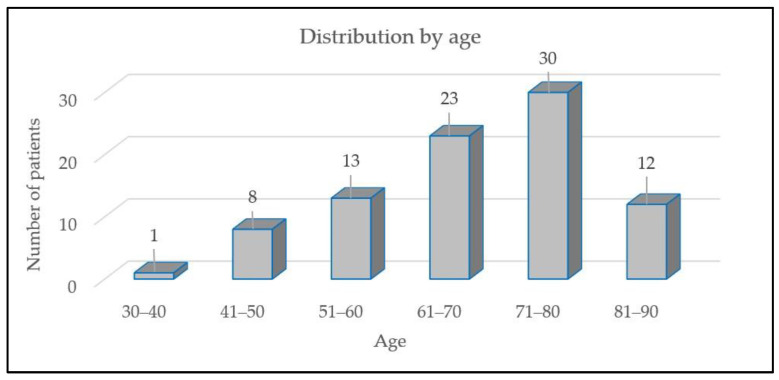
Distribution by age.

The current investigation comprised 87 patients, 31 female participants (35.63%) and 56 male participants (64.34%). The average age was 68 years, ranging from a minimum of 33 years to a maximum of 90 years (Figure 1). The mean BMI was 28.9 ± 5.6, with a range between 15.60 and 49.40. Of the total number of patients, 63 patients (72.41%) had fatal outcomes, while 24 patients (27.59%) survived. The typical duration of the stay in the intensive care unit was 14 days. Sepsis was prevalent in 57 patients (65.52%) and septic shock in 30 patients (34.48%).

In the study group, the prevalent underlying conditions and the infectious site varied, as detailed in Table 1. In the “Other” category, we included pathologies such as: secondary anemia, thrombocytopenia, chronic smoking, chronic alcohol intake, eschars, acid–base disorders, multiple organ dysfunction syndrome, hypovolemia, malnutrition, and electrolyte disorders, etc.

### 3.2. Comparison of Variables for the Entire Amount of Patients

**Figure 2:**
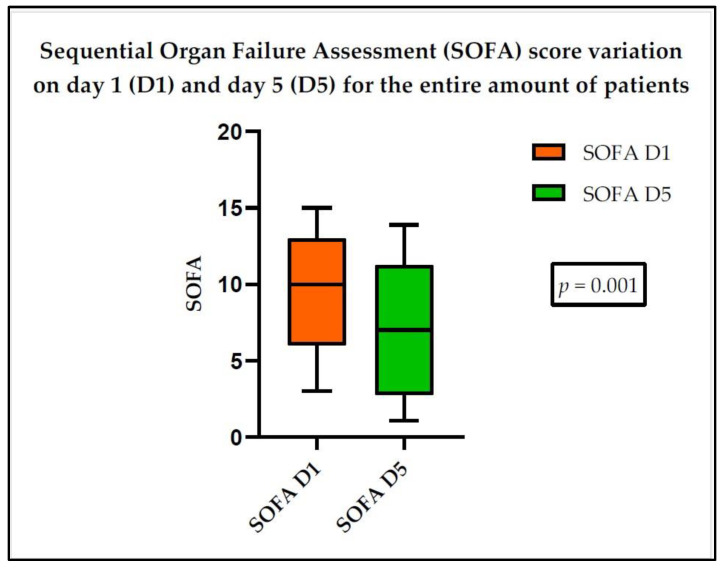
SOFA score variation, for the entire amount of patients, between day 1 and day 5.

Table S1 shows the studied parameters on day 1 and day 5. Day 1 is defined as the day of establishing the diagnosis of either sepsis or septic shock, according to the Sepsis-3 Consensus [4]. According to the analysed data, no significant differences were observed between day 1 and day 5, except for the SOFA score (Figure 2).

Table 2 presents an evaluation of the correlations between the subcategories of leukocytes and the inflammation markers. 

A statistically significant negative correlation was found between cytotoxic T lymphocytes and PD-1, and a positive correlation was found with PD-L1 on day 1. As for day 5, we observed a statistically significant negative correlation between helper T lymphocytes and the PD-L1 pathway, and a statistically significant positive correlation was found between cytotoxic T lymphocytes and PD-L1.

**Figure 3:**
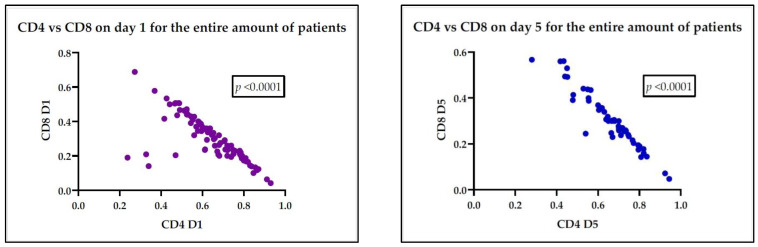
Statistically significant negative correlation between lymphocyte subtypes, Th CD4+ and Tc CD8+, on day 1—*p* < 0.0001 and day 5—*p* < 0.0001, after confirmation of either sepsis or septic shock.

Regarding the correlation between the subsets of lymphocytes, we found a statistically significant negative correlation on day 1 and day 5, represented visually in Figure 3.

Table 3 presents an evaluation of the correlations between the inflammation markers and the severity scores on day 1 and day 5, after confirmation of either sepsis or septic shock.

### 3.3. Comparison of Variables for the Sepsis and Septic Shock Groups

Table S2 compares the studied parameters between patients with sepsis and those who developed septic shock. 

**Figure 4:**
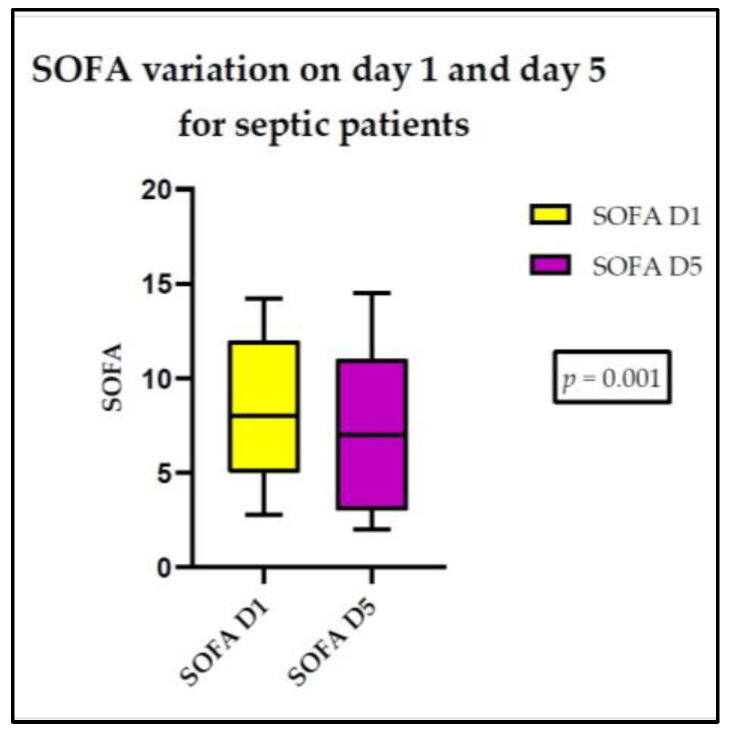
SOFA score variation for septic patients between day 1 and day 5.

**Figure 5:**
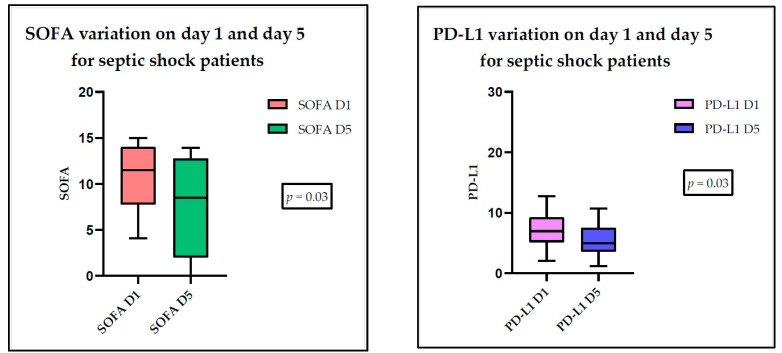
SOFA and PD-L1 score variation for septic shock patients between day 1 and day 5.

There was a significant variation in the SOFA score from day 1 to day 5, in both the sepsis and septic shock patients. We observed a statistically significant variation between the studied days for PD-L1 in septic shock patients. No significant differences were observed in regard to the other parameters under investigation (Figure 4 and Figure 5).

**Figure 6:**
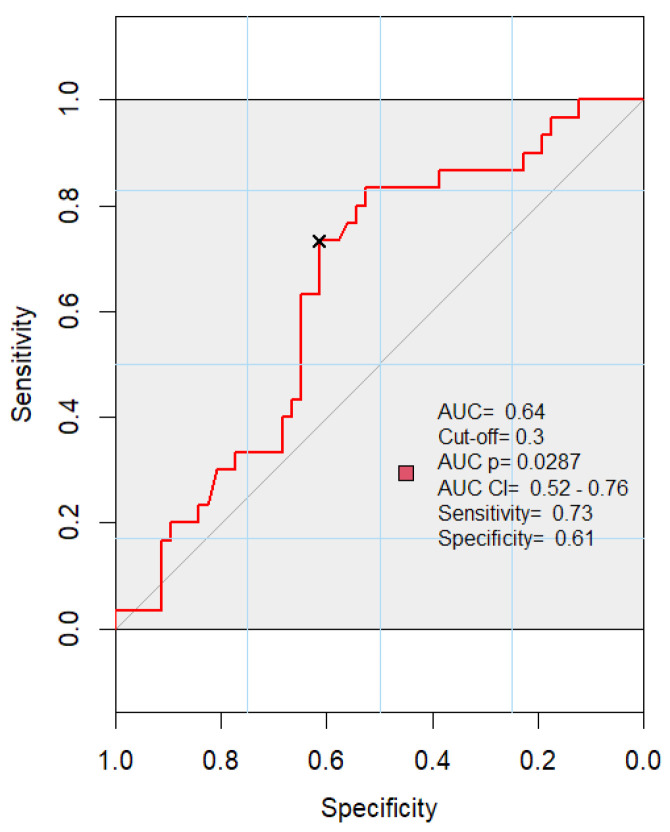
Receiver operating characteristic curve for Tc cells (CD8+) and septic shock (area under the curve: 0.64; *p* = 0.028).

Upon the examination of the ROC curves, it becomes apparent that only Tc cell (CD8+) values are significant in the early differentiation between sepsis and septic shock, as evidenced by the AUC (area under the curve) of 0.64, a cut-off value of 0.3, and a significance level of *p* = 0.028 on day 1 (Figure 6). Regarding survivors and non-survivors, the ROC curves showed little importance in discriminating between the two.

### 3.4. Comparison of Variables for the Survivor and Non-Survivor Groups

**Figure 7:**
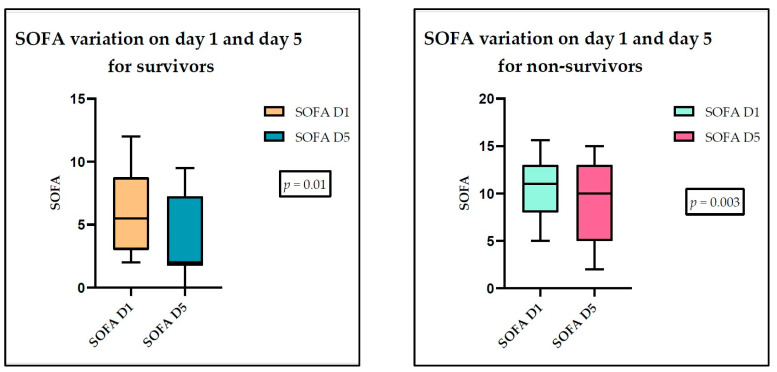
SOFA score variation between day 1 and day 5 for survivor and non-survivor groups.

Analyzing the data for survivors and non-survivors (Table S3), we found a statistically significant variation in the SOFA score for both groups of patients (Figure 7).

Table 4 and Table 5 present correlations for all the studied parameters on day 1 and day 5 for sepsis and septic shock patients.

### 3.5. Comparison of Variables for the Survivor and Non-Survivor Groups

Table 6 and Table 7 present correlations for all the studied parameters on day 1 and day 5 for survivor and non-survivor groups.

## 4. Discussion

Despite the significant financial burden associated with sepsis, the connection between morbidity and the site of infection remains unclear [19,20]. 

Our study found that pulmonary, abdominal, urinary tract, and cutaneous sites were predominant, while the prevalent underlying conditions were cardiovascular disease, renal disease, respiratory disease, neurological disease, and diabetes. The findings of our study align with those reported in the literature. A study conducted by V. Klastrup and colleagues, investigating the potential association between mortality and the site of infection, revealed that lung and gastrointestinal infections were prevalent [21]. Youli Chen and collaborators, in a 2022 study, assessed the link between the site of infection and mortality in patients with cancer and sepsis or septic shock and observed that lung, urinary tract, unspecified site, and gastrointestinal infections, were the most frequent, with gastrointestinal infection and pneumonia exhibiting the highest mortality rates [22].

Sepsis is regarded as being a biphasic disease, with both pro- and anti-inflammatory phases appearing almost synchronously. Since the point of transition from the aggressive pro-inflammatory phase to the anti-inflammatory phase is not exactly established in regard to the timeline of sepsis, immunological monitoring should focus on intricate biomarkers to capture this transition. We selected day 1 and day 5 as the study intervals, aligned with the diagnosis of sepsis or septic shock, as per the Sepsis-3 Consensus and IRIS-7 trial [4,23,24].

Severity scores remain a dependable tool in ICUs for assessing the prognosis of sepsis or septic shock, by gauging the disease severity, treatment response, and ICU mortality risk. However, the accuracy of these scores in predicting morbidity and mortality is still under debate, as they reflect the disease state at a specific moment during the patient’s ICU stay. Therefore, continuous re-evaluation of the SOFA and APACHE II score, either individually or in combination, is warranted [25]. Upon comparing the parameters on day 1 and day 5, we discerned a statistically significant alteration in the SOFA score, consistent with contemporary literature [4,26]. Notably, upon segregating the patients into survivors and non-survivors, and sepsis and septic shock groups, the correlation between the severity scores, SOFA and APACHE II, was statistically significant on both day 1 and day 5 for all the categories above, as well as in terms of the variation in the SOFA score from day 1 to day 5 for both sepsis and septic shock patients, providing indirect information about the multiple organ dysfunction that is secondary to apoptosis, in which septic and septic shock patients find themselves at a particular moment in the disease evolution. 

The prognostic significance of severity scores was augmented upon admission, influenced by the confluence of multiple comorbidities in patients, compounded by associated diseases in the decompensated state.

Nonetheless, these severity scores do not account for other factors, such as the patient’s medical history, lifestyle, medications received, or the quality of follow-up care, which are essential for estimating the long-term morbidity and mortality of sepsis or septic shock patients [27]. 

Recent years have seen a growing focus on strategies that can effectively alleviate the immunosuppressive condition in sepsis patients. A timely and precise evaluation of the immune status is essential for identifying immune dysfunction promptly in sepsis patients and for determining the appropriate timing for immunomodulatory therapy [3,28].

We found a statistically significant negative correlation between CD4+ and CD8+ T lymphocytes in both day 1 and day 5, suggesting a decrease in the value of both helper and cytotoxic lymphocytes, irrespective of sepsis or septic shock or the survival status. This correlation is attributed to profound immunosuppression and heightened inflammation, characteristic of the immunosenescent process. This process resembles inflammaging, wherein chronic, low-level sterile inflammation is integral to the aging process [29]. Known for their role in adaptive immunity, helper T lymphocytes activate B cells and cytotoxic T lymphocytes. Thus, although not statistically significant, we observed an increase in the value of CD4+ helper cells in septic patients, together with a decrease in CD8+ cytotoxic cells between day 1 and day 5 in sepsis patients and, in septic shock patients, we observed a slight decrease in CD4+ cells and an increase in the median value of CD8+ cells. The fluctuation in T lymphocytes plays a pivotal role in the immune response against sepsis; however, immunoparalysis can significantly compromise lymphocyte function, despite their numerical values falling within the normal range [24,30]. The results are supported by other recent studies that found a significantly reduced number of CD4+ cells in sepsis patients [15]. 

In sepsis, immunosuppression encompasses various cell types and characteristics, notably including heightened apoptosis of T and B cells, T cell exhaustion, and the diminished expression of activating cell-surface molecules. The apoptosis of immune cells is especially evident in CD4+ T cells, CD8+ T cells, B cells, natural killer cells, and follicular dendritic cells. Our study found decreased numbers of CD4+ and CD8+ T lymphocytes in both day 1 and day 5, results that are in accordance with recent literature, where postmortem examinations of patients who succumbed to sepsis revealed reduced populations of splenic T cells compared to those who passed away from non-infectious causes [9,31].

The depletion of follicular dendritic cells significantly hampers the function of T and B cells, leading to a CD4+ T cell deficit that impedes macrophage activation. CD8+ T cells, crucial for combating viral infections, exhibit reduced significance in the immune response of sepsis patients, primarily induced by bacterial infection [15,32,33]. 

Immune checkpoint inhibitory receptors found on immune cells initiate signalling pathways that promote immunosuppression. These molecules act as regulators, akin to brakes, controlling the adaptive immune response. PD-1, an immune checkpoint receptor, is pivotal in modulating the quantity and functional capacity of T cells. PD-L1 expression is noted on T cells, B cells, and antigen-presenting cells, as well as in certain non-lymphoid tissues. The binding of ligands to PD-1 receptors on T cells results in immune inhibition, suppressing T-cell activation and promoting apoptosis. Furthermore, PD-L1 is present in cardiac endothelial cells, placental tissues, and pancreatic islets, suggesting its involvement in immunological tolerance [32,34,35,36]. 

Our study found a correlation between Tc CD8+ cells and PD-1 and PD-L1 cells on day 1, as well as a correlation between Th CD4+ and Tc CD8+ lymphocytes and PD-L1 on day 5, providing proof that the enhanced expression of the PD-1/PD-L1 axis in sepsis induces immune suppression, by depleting lymphocyte subpopulations. By assessing the components of the PD-1/PD-L1 axis, alongside the severity scores, on the designated study days, we noted a positive correlation between both SOFA and APACHE II scores and PD-L1 levels on day 1. This suggests a potential unfavourable prognosis, given that PD-L1 gene expression is associated with inflammation, aligning with its function in inhibiting T cell activation [16,37]; thus, the use of blocking antibodies to impede the PD-1/PD-L1 axis has demonstrated efficacy in safeguarding against sepsis-induced immune suppression. This mechanism partly involves the inhibition of lymphocyte apoptosis and the reversal of monocyte dysfunction [24,38].

In our study, we assessed the influence of the components of the PD-1/PD-L1 axis on sepsis or septic shock. PD-1 modulates the quantity and functionality of T lymphocytes, while binding of the PD-L1 ligand determines the suppression of T lymphocyte activation and, thus, causes apoptosis. Thus, we found a statistically significant positive correlation between PD-1 and PD-L1 on day 1, as well as a statistically significant positive correlation between PD-L1 and the SOFA score on day 1 in septic shock patients. Our study reveals a statistically significant positive correlation between PD-L1 and the SOFA score in septic shock patients on day 1, reflecting the presence of multiple organ dysfunction and an immunosuppressed status, increasing the risk of mortality in these patients. 

Regarding non-survivors, we found stronger evidence to support immunosuppression, by observing a statistically significant positive correlation between CD4+ helper cells and PD-1 on day 1, which shifted to a statistically significant negative correlation on day 5 of study inclusion. This is in accordance with the study by Shao R. et al., which found increased expression of PD-L1 on the immune cells of septic shock patients that has been shown to elevate the mortality rate during the first 28 days of disease [39], as well as activating inhibitory immune checkpoints, metabolic disruptions, the impaired production of effector cytokines, decreased proliferation, and heightened vulnerability to apoptotic cell death [16]. The PD-1 pathway is crucial in regulating immune responses within host tissues, by facilitating the downregulation of inflammatory responses and restoring equilibrium in the immune system. Elevated PD-1 expression correlates with the activation of B cell receptors or T cell receptors in lymphocytes [40,41]; hence, we found a decrease in PD-1 on day 5 in non-survivor patients, together with CD4+ helper T cell immune exhaustion. This explains the negative impact of sepsis-derived changes on the outcome of patients. In 2018, Hu Y.M. et al., in an animal sepsis model, documented an elevation in PD-1 expression on peripheral T cells and PD-L1 on spleen B cells and monocytes [42]. 

An increased inflammatory response leading to immune suppression was observed in non-survivors, where a statistically significant negative correlation was found between CD8+ cytotoxic T cells and PD-1 and PD-L1 on day 1 of study inclusion. Although not statistically significant, we observed a negative correlation between these parameters on day 5. The increased expression of PD-L1 on day 5 suppresses the activation of CD8+ lymphocytes, leading to increased mortality and the development of secondary infections, which is in accordance with a previous study published in 2022 by Torres L.K. et al. [31]. 

We assessed the correlation between both severity scores, SOFA and APACHE II, and the PD-1/PD-L1 axis, in order to provide an extended picture of the organ dysfunction and immunological status of sepsis and septic shock patients, and we found a statistically significant positive correlation between PD-L1 and the severity scores on day 1 in non-survivor patients. This is in accordance with the study by Shao R. and collaborators, which found increased expression of PD-L1 on the immune cells of septic shock patients that has been shown to elevate the mortality rate during the first 28 days of disease [38]. The immune response and secondary immune paralysis related to sepsis are key elements in the patient’s progression. A dynamic assessment of the PD-1/PD-L1 axis, correlated with the severity scores, provides a more accurate overview of a patient with sepsis or septic shock and their outcome. In sepsis, obstructing the interaction between PD-1 on T cells and PD-L1 prevents T cell depletion, and correlates with enhanced microbial clearance and a reduced mortality rate [43,44].

The current study is subject to certain limitations. The relatively small sample size posed challenges in reaching definitive conclusions. Additionally, being a single-centre study introduced some bias in the assessment of the pathology. Moving forward, the authors aim to expand the study group and further assess the examined parameters in a larger cohort of patients.

## 5. Conclusions

The decrease in T lymphocyte subpopulations, Th CD4+ and Tc CD8+, was evident from day 1 of the confirmation of either sepsis or septic shock, prompting the inclusion of apoptosis as a key factor in the evolution of sepsis and septic shock. In septic shock, the increase in CD8+ cells between day 1 and day 5 revealed an aggressive pro-inflammatory status, correlated with the severity of the disease and high mortality.

The enhanced expression of the PD-1/PD-L1 axis diminishes costimulatory signaling, leading to a reduction in the capacity of T cell responses, lymphopenia, elevated mortality rates, and increased susceptibility to nosocomial infections. 

The correlation between the severity scores, SOFA and APACHE II, and the PD-1/PD-L1 axis provides an accurate overview of sepsis and septic shock and could be used as a mortality predictor for these patients. 

Further studies should focus on identifying the biomarkers associated with PD-1/PD-L1 expression and lymphocyte depletion, which could be used for prognostic purposes or to guide therapeutic decisions in sepsis.

## Figures and Tables

**Table 1:** Pathology and infectious site for the entire amount of patients.

Underlying Conditions	Number of Patients	%	Infectious Site	Number of Patients	%
Cardiovascular disease	73	83.91	Pulmonary	51	58.6
Renal disease	58	69.88	Abdominal	30	34.5
Respiratory disease	55	63.22	Urinary tract	10	11.5
Neurological disease	42	48.28	Cutaneous	7	8.0
Diabetes	27	31.03	Thoracic cavity	1	1.1
Trauma	7	8.05	Soft tissue	1	1.1
Other	85	97.70	Unidentified	1	1.1

**Table 2:** Correlations between PD-1 and PD-L1 and the leukocyte subcategories on day 1 and day 5 for the entire amount of patients.

Parameter (on Day 1)	PD-1 (ng/mL)	PD-L1 (ng/mL)
Th cells (CD4+) %	r = 0.1941 (−0.03038 to 0.4000) ^a^ *p* = 0.08	r = 0.1378 (−0.09043 to 0.3523) *p* ^b^ = 0.24
Tc cells (CD8+) %	r = −0.2545 (−0.4520 to −0.03317) ^a^ *p* = 0.02	r =−0.2263 (−0.4353 to 0.005916) ^a^ *p* = 0.04
**Parameter (on day 5)**	**PD-1 (ng/mL)**	**PD-L1 (ng/mL)**
Th cells (CD4+) %	r = −0.1661 (−0.4397 to 0.1357) ^a^ *p* =0.27	r = −0.3306 (−0.5793 to −0.02552) *p* ^b^ = 0.03
Tc cells (CD8+) %	r = 0.1366 (−0.1652 to 0.4150) ^a^ *p* = 0.36	rho = 0.3982 (0.1032 to 0.6288) *p* ^b^ = 0.0099
**Parameter**	**Th cells (CD4+) % on day 1**	**Th cells (CD4+) % on day 5**
Tc cells (CD8+) %	r = −0.7951 (−0.8631 to −0.6988) ^a^ *p* ≤ 0.0001	r = −0.9558 (−0.9745 to −0.9238) *p* ^b^ ≤ 0.0001

^a^ Spearman test, ^b^ Pearson test. Th cells: T helper lymphocytes, Tc cells: T cytotoxic lymphocytes, PD-1: programmed cell death protein 1, PD-L1: programmed death ligand 1.

**Table 3:** Correlations between PD-1 and PD-L1 and the severity scores on day 1 and day 5 for the entire amount of patients.

Parameter (on Day 1)	PD-1 (ng/mL)	PD-L1 (ng/mL)
SOFA	r = 0.08570 (−0.1402 to 0.3031) ^a^ *p* = 0.44	r= 0.3511 (0.1365 to 0.5343) *p* ^b^ = 0.001
APACHE II	r = 0.1346 (−0.09132 to 0.3474) ^a^ *p* = 0.2278	r = 0.2822 (0.06065 to 0.4773) ^a^ *p* = 0.01
**Parameter (on day 5)**	**PD-1 (ng/mL)**	**PD-L1 (ng/mL)**
SOFA	r = 0.06812 (−0.2350 to 0.3592) ^a^ *p* = 0.65	r = 0.03967 (−0.2752 to 0.3469) *p* ^b^ = 0.80
APACHE II	r = 0.1019 (−0.2026 to 0.3885) ^a^ *p* = 0.5	rho = 0.1580 (−0.1615 to 0.4475) *p* ^b^ = 0.33
**Parameter**	**SOFA on day 1**	**SOFA on day 5**
APACHE II	r = 0.7394 (0.6263 to 0.8220) *p* ^b^ ≤ 0.0001	r = 0.7726 (0.6297 to 0.8649) *p* ^b^ ≤ 0.0001

^a^ Spearman test, ^b^ Pearson test. PD-1: programmed cell death protein 1, PD-L1: programmed death ligand 1, SOFA: Sequential Organ Failure Assessment, APACHE II: Acute Physiology and Chronic Health Evaluation.

**Table 4:** Correlations for sepsis patients on day 1 and day 5.

		Tc Cells (CD8+) %	PD-1, ng/mL	PD-L1, ng/mL	SOFA	APACHE II
Th cells (CD4+) %	Day 1	r = −0.8654 (−0.9188 to −0.7809) *p* ^b^ < 0.0001	r = 0.1386 (−0.1449 to 0.4010) ^a^ *p* = 0.3224	r = 0.1374 (−0.1496 to 0.4030) *p* ^b^ = 0.3466	r = 0.08207 (−0.1824 to 0.3355) *p* ^b^ = 0.5439	r = 0.09483 (−0.1699 to 0.3468) *p* ^b^ = 0.4829
	Day 5	r = −0.9776 (−0.9887 to −0.9557) *p* ^b^ < 0.0001	r = −0.2788 (−0.5881 to 0.1016) ^a^ *p* = 0.1357	r = −0.001538 (−0.4068 to 0.4042) ^a^ *p* = 0.9942	r = 0.01774 (−0.3330 to 0.3642) *p* ^b^ = 0.9232	r = 0.01039 (−0.3395 to 0.3578) *p* ^b^ = 0.9550
Tc cells (CD8+) %	Day 1		r = −0.1885 (−0.4431 to 0.09433) ^a^ *p* = 0.1765	r = −0.2057 (−0.4603 to 0.08011) *p* ^b^ = 0.1562	r = −0.1455 (−0.3913 to 0.1196) *p* ^b^ = 0.2801	r = −0.1744 (−0.4161 to 0.09023) *p* ^b^ = 0.1944
	Day 5		r = 0.2487 (−0.1336 to 0.5665) ^a^ *p* = 0.1852	r = 0.1078 (−0.3113 to 0.4918) ^a^ *p* = 0.6081	r = −0.08839 (−0.4240 to 0.2686) *p* ^b^ = 0.6305	r = −0.06552 (−0.4050 to 0.2898) *p* ^b^ = 0.7216
PD-1, ng/mL	Day 1			r = 0.002837 (−0.2958 to 0.3010) ^a^ *p* = 0.9851	r = −0.004792 (−0.2823 to 0.2734) ^a^ *p* = 0.9728	r = 0.2031 (−0.07920 to 0.4553) ^a^ *p* = 0.1446
	Day 5			r = −0.06957 (−0.4700 to 0.3546) ^a^ *p* = 0.7467	r = −0.04681 (−0.4096 to 0.3288) ^a^ *p* = 0.8060	r = 0.05985 (−0.3171 to 0.4205) ^a^ *p* = 0.7534
PD-L1, ng/mL	Day 1				r = 0.2086 (−0.07714 to 0.4626) *p* ^b^ = 0.1504	r = 0.2338 (−0.05075 to 0.4832) *p* ^b^ = 0.1059
	Day 5				r = 0.003482 (−0.4026 to 0.4084) ^a^ *p* = 0.9868	r = 0.2367 (−0.1868 to 0.5859) ^a^ *p* = 0.2547
SOFA	Day 1					r = 0.7225 (0.5691 to 0.8273) *p* ^b^ < 0.0001
	Day 5					r = 0.7294 (0.5193 to 0.8563) *p* ^b^ < 0.0001

^a^ Spearman test, ^b^ Pearson test. Th cells: T helper lymphocytes, Tc cells: T cytotoxic lymphocytes, PD-1: programmed cell death protein 1, PD-L1: programmed death ligand 1, SOFA: Sequential Organ Failure Assessment, APACHE II: Acute Physiology and Chronic Health Evaluation.

**Table 5:** Correlations for septic shock patients on day 1 and day 5.

		Tc Cells (CD8+) %	PD-1, ng/mL	PD-L1, ng/mL	SOFA	APACHE II
Th cells (CD4+) %	Day 1	r = −0.4830 (−0.7183 to −0.1486) *p* ^b^ = 0.0069	r = 0.2340 (−0.1561 to 0.5609) ^a^ *p* = 0.2219	r = 0.06571 (−0.3224 to 0.4349) *p* ^b^ = 0.7447	r = 0.03528 (−0.3292 to 0.3906) *p* ^b^ = 0.8532	r = 0.05343 (−0.3129 to 0.4059) *p* ^b^ = 0.7792
	Day 5	r = −0.9042 (−0.9653 to −0.7491) *p* ^b^ < 0.0001	r = −0.03431 (−0.5180 to 0.4660) ^a^ *p* = 0.8984	r = −0.4075 (−0.7514 to 0.1105) *p* ^b^ = 0.1172	r = 0.07724 (−0.4351 to 0.5518) *p* ^b^ = 0.7762	r = 0.001199 (−0.4948 to 0.4966) *p* ^b^ = 0.9965
Tc cells (CD8+) %	Day 1		r = −0.3345 (−0.6313 to 0.04784) ^a^ *p* = 0.0761	r = −0.04149 (−0.4150 to 0.3439) *p* ^b^ = 0.8372	r = −0.1220 (−0.4620 to 0.2492) *p* ^b^ = 0.5206	r = 0.01866 (−0.3439 to 0.3764) *p* ^b^ = 0.9220
	Day 5		r = −0.02696 (−0.5126 to 0.4718) ^a^ *p* = 0.9209	r = 0.4329 (−0.08000 to 0.7645) *p* ^b^ = 0.0940	r = −0.09829 (−0.5664 to 0.4178) *p* ^b^ = 0.7173	r = −0.03894 (−0.5245 to 0.4658) *p* ^b^ = 0.8861
PD-1, ng/mL	Day 1			r = 0.4566 (0.07218 to 0.7230) ^a^ *p* = 0.0190	r = 0.1846 (−0.2060 to 0.5244) ^a^ *p* = 0.3378	r = 0.06407 (−0.3199 to 0.4300) ^a^ *p* = 0.7413
	Day 5			r = 0.3088 (−0.2359 to 0.7059) ^a^ *p* = 0.2440	r = 0.3333 (−0.2099 to 0.7193) ^a^ *p* = 0.2058	r = 0.1372 (−0.3983 to 0.6029) ^a^ *p* = 0.6101
PD-L1, ng/mL	Day 1				r = 0.4909 (0.1363 to 0.7340) *p* ^b^ = 0.0093	r = 0.3373 (−0.04903 to 0.6358) *p* ^b^ = 0.0854
	Day 5				r = 0.1272 (−0.4119 to 0.6003) *p* ^b^ = 0.6515	r = 0.1370 (−0.4036 to 0.6067) *p* ^b^ = 0.6263
SOFA	Day 1					r = 0.8070 (0.6298 to 0.9043) *p* ^b^ < 0.0001
	Day 5					r = 0.8433 (0.5972 to 0.9443) *p* ^b^ < 0.0001

^a^ Spearman test, ^b^ Pearson test. Th cells: T helper lymphocytes, Tc cells: T cytotoxic lymphocytes, PD-1: programmed cell death protein 1, PD-L1: programmed death ligand 1, SOFA: Sequential Organ Failure Assessment, APACHE II: Acute Physiology and Chronic Health Evaluation.

**Table 6:** Correlations for survivors on day 1 and day 5.

		Tc Cells (CD8+) %	PD-1, ng/mL	PD-L1, ng/mL	SOFA	APACHE II
Th cells (CD4+) %	Day 1	r = −0.9368 (−0.9726 to −0.8575) *p* ^b^ < 0.0001	r = −0.2344 (−0.5910 to 0.1988) ^a^ *p* = 0.2703	r = 0.1030 (−0.3679 to 0.5319) ^a^ *p* = 0.6655	r = 0.07439 (−0.3392 to 0.4639) *p* ^b^ = 0.7298	r = −0.09935 (−0.4834 to 0.3167) *p* ^b^ = 0.6442
	Day 5	r = −0.9788 (−0.9922 to −0.9426) *p* ^b^ < 0.0001	r = 0.05296 (−0.4547 to 0.5346) *p* ^b^ = 0.8456	r = −0.06786 (−0.5720 to 0.4735) ^a^ *p* = 0.8124	r = −0.1823 (−0.6600 to 0.4004) ^a^ *p* = 0.5303	r = −0.2830 (−0.7074 to 0.2913) *p* ^b^ = 0.3268
Tc cells (CD8+) %	Day 1		r = 0.1723 (−0.2602 to 0.5472) ^a^ *p* = 0.4207	r = −0.07446 (−0.5110 to 0.3926) ^a^ *p* = 0.7550	r = −0.1931 (−0.5534 to 0.2280) *p* ^b^ = 0.3659	r = 0.008656 (−0.3961 to 0.4106) *p* ^b^ = 0.9680
	Day 5		r = −0.01212 (−0.5048 to 0.4865) *p* ^b^ = 0.9645	r = 0.1893 (−0.3722 to 0.6493) ^a^ *p* = 0.4983	r = 0.1170 (−0.4549 to 0.6206) ^a^ *p* = 0.6894	r = 0.3229 (−0.2506 to 0.7286) *p* ^b^ = 0.2602
PD-1, ng/mL	Day 1			r = −0.1835 (−0.5882 to 0.2948) ^a^ *p* = 0.4388	r = 0.02186 (−0.3957 to 0.4319) ^a^ *p* = 0.9193	r = −0.1161 (−0.5057 to 0.3129) ^a^ *p* = 0.5892
	Day 5			r = 0.05934 (−0.4998 to 0.5836) ^a^ *p* = 0.8438	r = 0.2519 (−0.3633 to 0.7141) ^a^ *p* = 0.4044	r = 0.006637 (−0.5463 to 0.5556) *p* ^b^ = 0.9828
PD-L1, ng/mL	Day 1				r = −0.05898 (−0.4994 to 0.4056) ^a^ *p* = 0.8049	r = −0.07472 (−0.5111 to 0.3923) ^a^ *p* = 0.7542
	Day 5				r = −0.1015 (−0.6495 to 0.5159) ^a^ *p* = 0.7583	r = −0.2050 (−0.7067 to 0.4339) ^a^ *p* = 0.5201
SOFA	Day 1					r = 0.7352 (0.4718 to 0.8782) *p* ^b^ < 0.0001
	Day 5					r = 0.8397 (0.5452 to 0.9497) *p* ^b^ = 0.0003

^a^ Spearman test, ^b^ Pearson test. Th cells: T helper lymphocytes, Tc cells: T cytotoxic lymphocytes, PD-1: programmed cell death protein 1, PD-L1: programmed death ligand 1, SOFA: Sequential Organ Failure Assessment, APACHE II: Acute Physiology and Chronic Health Evaluation.

**Table 7:** Correlations for non-survivors on day 1 and day 5.

		Tc Cells (CD8+) %	PD-1, ng/mL	PD-L1, ng/mL	SOFA	APACHE II
Th cells (CD4+) %	Day 1	r = −0.7114 (−0.8155 to −0.5629) *p* ^b^ < 0.0001	r = 0.3463 (0.08886 to 0.5603) ^a^ *p* = 0.0078	r = 0.1843 (−0.08264 to 0.4265) *p* ^b^ = 0.1740	r = 0.1454 (−0.1062 to 0.3795) *p* ^b^ = 0.2556	r = 0.1835 (−0.06737 to 0.4125) *p* ^b^ = 0.1501
	Day 5	r = −0.9461 (−0.9730 to −0.8939) *p* ^b^ < 0.0001	r = −0.4123 (−0.6749 to −0.05702) ^a^ *p* = 0.0212	r = −0.2116 (−0.5536 to 0.1915) *p* ^b^ = 0.2995	r = 0.02640 (−0.3146 to 0.3613) *p* ^b^ = 0.8822	r = 0.01088 (−0.3285 to 0.3478) *p* ^b^ = 0.9513
Tc cells (CD8+) %	Day 1		r = −0.3873 (−0.5920 to −0.1357) ^a^ *p* = 0.0027	r = −0.2790 (−0.5049 to −0.01736) *p* ^b^ = 0.0373	r = −0.2283 (−0.4506 to 0.02059) *p* ^b^ = 0.0719	r = −0.1836 (−0.4126 to 0.06722) *p* ^b^ = 0.1498
	Day 5		r = 0.3414 (−0.02566 to 0.6273) ^a^ *p* = 0.0602	r = 0.2440 (−0.1584 to 0.5768) *p* ^b^ = 0.2297	r = −0.1121 (−0.4338 to 0.2350) *p* ^b^ = 0.5280	r = −0.1009 (−0.4246 to 0.2457) *p* ^b^ = 0.5703
PD-1, ng/mL	Day 1			r = 0.2528 (−0.02984 to 0.4980) ^a^ *p* = 0.0706	r = 0.1357 (−0.1347 to 0.3873) ^a^ *p* = 0.3097	r= 0.2432 (−0.02392 to 0.4779) ^a^ *p* = 0.0658
	Day 5			r = 0.03316 (−0.3693 to 0.4251) ^a^ *p* = 0.8722	r = 0.05831 (−0.3005 to 0.4026) ^a^ *p* = 0.7472	r = 0.1770 (−0.1872 to 0.4985) ^a^ *p* = 0.3243
PD-L1, ng/mL	Day 1				r = 0.4850 (0.2545 to 0.6633) *p* ^b^ = 0.0002	r = 0.3710 (0.1198 to 0.5776) *p* ^b^ = 0.0049
	Day 5				r = −0.004608 (−0.3770 to 0.3691) *p* ^b^ = 0.9814	r = 0.1172 (−0.2676 to 0.4697) *p* ^b^ = 0.5526
SOFA	Day 1					r = 0.6471 (0.4756 to 0.7712) *p* ^b^ < 0.0001
	Day 5					r = 0.6939 (0.4733 to 0.8326) *p* ^b^ < 0.0001

^a^ Spearman test, ^b^ Pearson test. Th cells: T helper lymphocytes, Tc cells: T cytotoxic lymphocytes, PD-1: programmed cell death protein 1, PD-L1: programmed death ligand 1, SOFA: Sequential Organ Failure Assessment, APACHE II: Acute Physiology and Chronic Health Evaluation.

## Data Availability

The data generated in the present study may be requested from the corresponding author.

## Supplementary Materials

The following supporting information can be downloaded at https://www.mdpi.com/article/10.3390/medicina60071174/s1, Table S1: The studied parameters on day 1 and day 5 for the entire amount of patients (median value and interquartile range—IQR). Table S2: The studied parameters in sepsis versus septic shock on day 1 and day 5 (median value and IQR). Table S3: Comparison of the studied parameters between survivors and non-survivors on day 1 and day 5 (median value and IQR).
