# Supplementary material for: The Role of Programmed Cell Death 1/Programmed Death Ligand 1 (PD-1/PD-L1) Axis in Sepsis-Induced Apoptosis
Source: Medicina (Kaunas). 2024 Jul 19;60(7):1174. doi: 10.3390/medicina60071174 (PMC11278887; doi:10.3390/medicina60071174)
Supplement: Supplementary file 1 [file medicina-60-01174-s001.zip › medicina-3069233-supplementary.pdf]

Supplementary Material for "The role of Programmed cell death-1/Programmed death ligand 1 (PD-1/PD-L1) axis in sepsis-induced apoptosis"

Table S1. The studied parameters on day 1 and day 5 for the entire lot of patients (median value and interquartile range – IQR).

**Parameter**	**Day 1**	**Day 5**	***p* value**
Th cells (CD4+), %	63 (21)	66.7 (18.6)	0.45 [a]
Tc cells (CD8+), %	29.3 (19.6)	30 (14.9)	0.83 [a]
PD-1, ng/ml	0.18 (0.16)	0.16 (0.17)	0.47 [a]
PD-L1, ng/ml	6.29 (4.95)	5.64 (3.61)	0.79 [a]
SOFA	10 (7)	7 (8.5)	0.001 [a]
APACHE II	23 (12)	19 (15.75)	0.11 [a]

[a] Wilcoxon. Th cells: T helper lymphocytes, Tc cells: T cytotoxic lymphocytes, PD-1: Programmed cell death protein 1, PD-L1: Programmed death ligand 1, SOFA: Sequential Organ Failure Assessment, APACHE II: Acute Physiology and Chronic Health Evaluation.

Table S2. The studied parameters in sepsis versus septic shock on day 1 and day 5 (median value and IQR).

**Parameter**	**Sepsis**		***p* value**	**Septic shock**		***p* value**
	**Day 1**	**Day 5**		**Day 1**	**Day 5**	
Th cells (CD4+), %	60.4 (22.45)	65 (19.2)	0.74 [a]	67.95 (15.72)	67.8 (15.4)	0.26 [a]
Tc cells (CD8+), %	34.5 (21.85)	30 (17.6)	0.86 [a]	24.1 (12.6)	26 (9)	0.54 [a]
PD-1, ng/ml	0.16 (0.13)	0.14 (0.12)	0.33 [a]	0.21 (0.15)	0.2 (0.22)	0.99 [a]
PD-L1, ng/ml	5.52 (4.95)	5.82 (3.9)	0.86 [a]	6.96 (4.13)	4.99 (3.95)	0.03 [a]
SOFA	8 (7)	7 (8)	0.001 [a]	11.5 (6.25)	8.5 (10.75)	0.03 [a]
APACHE II	22 (12)	19 (11.5)	0.07 [a]	26.5 (15)	20.5 (23)	0.65 [a]

[a] Wilcoxon. Th cells: T helper lymphocytes, Tc cells: T cytotoxic lymphocytes, PD-1: Programmed cell death protein 1, PD-L1: Programmed death ligand 1, SOFA: Sequential Organ Failure Assessment, APACHE II: Acute Physiology and Chronic Health Evaluation.

Table S3. Comparison of the studied parameters between survivors and non-survivors on day 1 and day 5 (median value and IQR).

**Parameter**	**Survivors**		***p* value**	**Non-survivors**		***p* value**
	**Day 1**	**Day 5**		**Day 1**	**Day 5**	
Th cells (CD4+), %	66.35 (15.9)	65.35 (21.45)	0.42 [a]	62.2 (22.7)	67.9 (18.43)	0.97 [a]
Tc cells (CD8+), %	28 (18.43)	30.6 (19.65)	0.26 [a]	29.3 (22.4)	30 (12.8)	0.9 [a]
PD-1, ng/ml	0.17 (0.14)	0.18 (0.29)	0.86 [a]	0.18 (0.16)	0.15 (0.13)	0.29 [a]
PD-L1, ng/ml	5.17 (3.71)	5.24 (2.65)	0.58 [a]	6.96 (5.52)	5.81 (5.22)	0.32 [a]
SOFA	5.5 (5.75)	2 (5.5)	0.01 [a]	11 (5)	10 (8)	0.003 [a]
APACHE II	13.5 (11)	10.5 (10.25)	0.24 [a]	27 (11)	22.5 (15.75)	0.39 [a]

[a] Wilcoxon. Th cells: T helper lymphocytes, Tc cells: T cytotoxic lymphocytes, PD-1: Programmed cell death protein 1, PD-L1: Programmed death ligand 1, SOFA: Sequential Organ Failure Assessment, APACHE II: Acute Physiology and Chronic Health Evaluation.
